# Vibrating barrier: a novel device for the passive control of structures under ground motion

**DOI:** 10.1098/rspa.2015.0075

**Published:** 2015-07-08

**Authors:** P. Cacciola, A. Tombari

**Affiliations:** School of Environment and Technology, University of Brighton, Brighton BN2 4GJ, UK

**Keywords:** vibration control, ground motion waves, structure–soil–structure interaction, vibrating barrier

## Abstract

A novel device, called vibrating barrier (ViBa), that aims to reduce the vibrations of adjacent structures subjected to ground motion waves is proposed. The ViBa is a structure buried in the soil and detached from surrounding buildings that is able to absorb a significant portion of the dynamic energy arising from the ground motion. The working principle exploits the dynamic interaction among vibrating structures due to the propagation of waves through the soil, namely the structure–soil–structure interaction. The underlying theoretical aspects of the novel control strategy are scrutinized along with its numerical modelling. Closed-form solutions are also derived to design the ViBa in the case of harmonic excitation. Numerical and experimental analyses are performed in order to investigate the efficiency of the device in mitigating the effects of ground motion waves on the structural response. A significant reduction in the maximum structural acceleration of 87% has been achieved experimentally.

## 1. Introduction

Control of building vibrations is crucial for structural safety and avoids the unexpected behaviours that lead to rapid deterioration or collapse of a structure. Various sources of vibrations can affect the structure, including human activities such as road traffic, high-speed trains, large machinery, rock drilling and blasting or natural disturbances such as wind gusts, ocean waves and earthquakes. Strategies for vibration control are based on the modification of the dynamic structural characteristics by: (i) increasing the dissipative properties of the structure, (ii) altering its rigidity for inducing the shift of the structural frequencies, or (iii) adding resonant devices able to absorb part of the structural vibrations. In civil engineering, devices for the passive control of ground motion waves are widely used since they do not require an external power source to operate; a few examples of passive control devices are viscous damper, tuned mass damper, tuned liquid damper, base isolation and dissipative bracing systems. These vibration control systems are successfully employed in the design of new structures; on the other hand, they are rarely used for protecting existing buildings, as they generally require substantial alteration of the original structure. In the case of heritage buildings and critical facilities or urban areas, especially in developing countries, these traditional localized solutions might become impractical. Therefore, alternative non-localized solutions represent a reliable strategy for this challenge.

In this regard, very few attempts have been made to investigate non-local strategies to ensure the safety of existing buildings, infrastructures and critical facilities. Namely, trench barriers or sheet-pile walls in the soil have been investigated for altering the displacement field based on the reflection, scattering and diffraction of dynamic surface waves (e.g. [1–5]). These attempts, even if mainly limited to the reduction of surface waves, highlighted the importance of focusing on the soil instead of the structure itself.

During the last two decades, studies on the site–city interaction [6–8] highlighted a substantial change in the ground motion wave field and the consequent dynamic response of buildings in an urban environment. Remarkably, Kham *et al.* [8] showed that the energy of ground motion at the free field in a city is reduced by up to 50% due to the perturbation induced by resonant buildings. The reasons for this phenomenon governing the site–city effects are based on the well-known structure–soil–structure interaction (SSSI) [9,10]. Warburton *et al.* [9] studied the dynamic response of two rigid masses in an elastic subspace, showing the influence of one mass with respect to the other. Luco & Contesse [10] studied the dynamic interaction between two parallel infinite shear walls placed on rigid foundations and forced by a vertically incident shear (SH) wave. Wong & Trifunac [11] extended the previous case for non-vertically incident plane SH waves by investigating the significance of the angle of incidence. A recent review of the SSSI problem can be found in Lou *et al.* [12].

**Figure 1.**
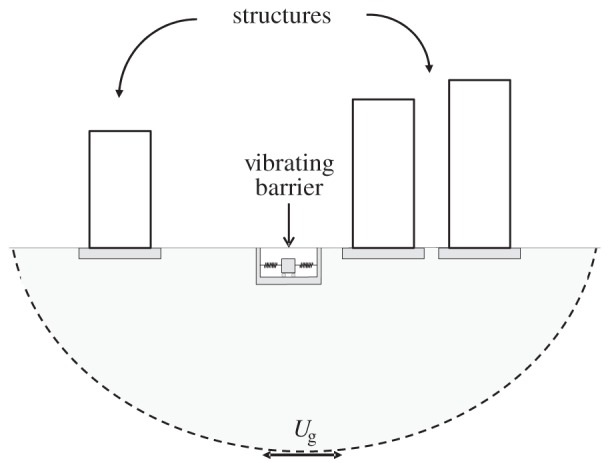
Schematic of the proposed strategy for the seismic protection of cluster of structures thought the novel ViBa.

The benefits arising from the presence of other buildings in reducing structural vibrations have not yet been exploited as a tool for seismic vibration control. This paper introduces for the first time a novel device, herein called the vibrating barrier (ViBa), that aims to reduce the vibrations of structures from ground motion waves by exploiting the SSSI phenomenon. Figure 1 illustrates the physical problem investigated in this paper: a cluster of buildings subjected to base excitation along with the proposed device, the ViBa, embedded in the soil for absorbing the input energy, reducing therefore damage and failures of the adjacent structures. The ViBa is a structure buried in the soil and detached from the surrounding buildings. It consists of an embedded foundation containing an internal oscillator unit that, if tuned appropriately, is able to absorb a significant part of the dynamic energy that would otherwise affect the structures. In order to study this novel vibration control strategy, a discrete model is first derived.

Discrete solutions to the SSSI problems using rigorous analytical formulations are available in the literature [13–16]. Kobori *et al.* [13] defined a multi-spring–mass system for investigating the dynamic coupling of two adjacent square superficial foundations. Mulliken & Karabalis [14] defined a simple discrete model for predicting the dynamic interaction between adjacent rigid, surface foundations supported by a homogeneous, isotropic and linear elastic half-space. Recently, Alexander *et al.* [15] developed a discrete model to study the SSSI problem of surface foundations by considering stochastic ground motion excitation; Aldaikh *et al.* [16] extended the work of Alexander *et al.* [15] to the case of three buildings with validation of the discrete theoretical model by means of experimental shake table testing.

Based on the same principles, the effects of the soil on the structures, i.e. the soil–structure interaction (SSI) as well as the SSSI and the ViBa–SSI, are taken into account in this paper by means of linear elastic springs, as in the conventional Winkler approach for a linear elastic soil medium.

The simplified model is able to capture the main effects of the interaction phenomena of the soil, as shown in the comparison with more advanced finite-element method (FEM)/boundary element method (BEM) numerical solutions in a model of a nuclear reactor. Moreover, experimental results showed a remarkable reduction in terms of maximum acceleration of 87% of a structure controlled by the ViBa prototype.

## 2. Governing equations of the structure–soil–structure interaction

**Figure 2.**
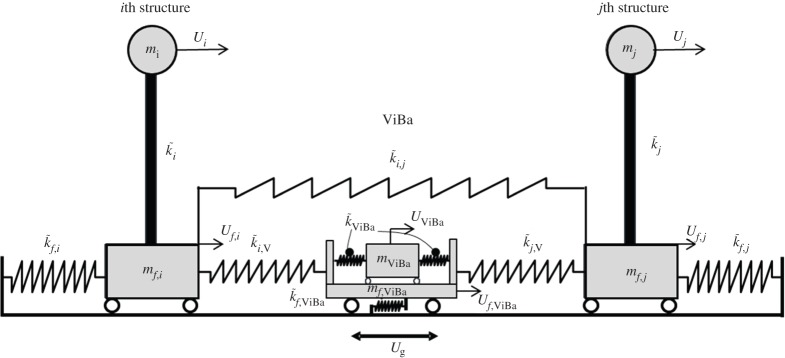
Discrete model adopted for the study of vibration control of two structures through the ViBa.

Consider the global system depicted in figure 1 under the ground excitation defined by the ground displacement *U*_*g*_. The proposed ViBa is also included with the aim of reducing the vibration of the surrounding buildings. In this regard, a mechanical model able to describe the interaction phenomenon is derived first. Figure 2 shows the mechanical relations of the *i*th and *j*th structures coupled with the ViBa. Each building is modelled as a 2 d.f. system with one translational d.f. at the top of the building and one at the foundation level, i.e. *U*_*i*_ and *U*_*f*,*i*_ for *i*=1,…,*n* (where *n* is the number of surrounding buildings). The ViBa is modelled as an internal unit device included in a rigid box foundation and globally described by the 2 d.f., *U*_ViBa_ and *U*_*f*,ViBa_. The dynamic governing equations of the global system are derived in terms of absolute displacement, as conventional in SSI, namely the dynamics of the problem take the form

**Figure 3.**
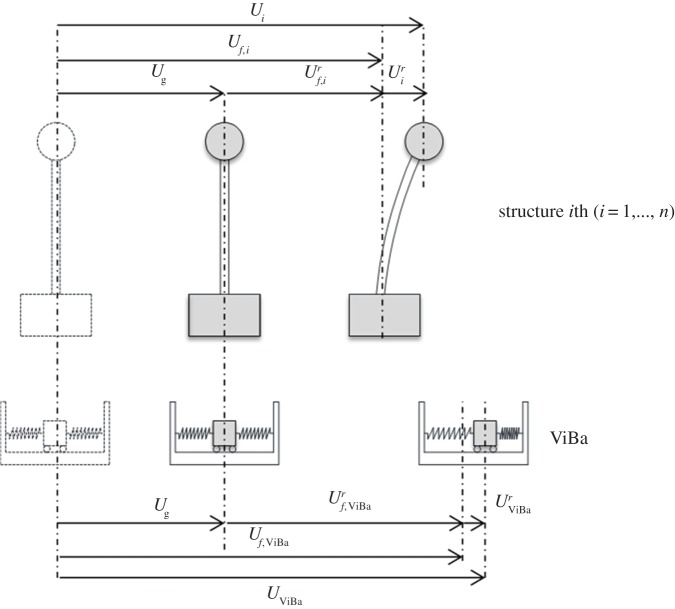
Definition of the kinematics quantities used in the formulation.

2.1 $$(\tilde{\text{K}}-\omega^{2}\text{M})\text{U}(\omega )=\text{Q}\;U_{g}(\omega ),$$

where **U**(*ω*) is the absolute displacement formulated in the frequency domain (*ω* is the circular frequency) in which the components are ordered as follows:

2.2 $${\text{U}}^{T}(\omega )=[\begin{array}{ccccccc} U_{i}(\omega ) & U_{f,i}(\omega ) & \cdots & U_{n}(\omega ) & U_{f,n}(\omega ) & U_{\mathrm{ViBa}}(\omega ) & U_{f,\mathrm{ViBa}}(\omega ) \end{array}],$$

where T indicates the transpose operator; for the sake of clarity the kinematics relations among the displacement components of the *i*th structure and of the ViBa are indicated in figure 3. In equation (2.1), **M** is the real global mass matrix and $\tilde{\text{K}}$ is the complex global stiffness matrix; note that the symbol $\tilde{\text{K}}$ is used on the stiffness quantities to emphasize the hysteretic damping model adopted in the paper (e.g. [17]).

In addition, the matrices of the global system are partitioned in the sub-matrices defined for the individual buildings and the ViBa; therefore, the global mass matrix is stated as follows:

2.3 $$\text{M}=\left[\begin{array}{ccc} \begin{array}{cc} {\text{M}}_{1} & 0\\ 0 & {\text{M}}_{i} \end{array} & \cdots & \begin{array}{cc} 0 & 0\\ 0 & 0 \end{array}\\ \vdots & \ddots & \vdots \\ \begin{array}{cc} 0 & 0\\ 0 & 0 \end{array} & \cdots & \begin{array}{cc} {\text{M}}_{n} & 0\\ 0 & {\text{M}}_{V} \end{array} \end{array}\right],$$

in which the *i*th sub-block is defined as

2.4 $${\text{M}}_{i}=\left[\begin{array}{cc} m_{i} & 0\\ 0 & m_{f,i} \end{array}\right],$$

where *m*_*i*_ is the mass of the *i*th structure and *m*_*f*,*i*_ is the mass of the *i*th foundation, while **M**_V_ is the mass matrix of the ViBa given by

2.5 $${\text{M}}_{V}=\left[\begin{array}{cc} m_{\mathrm{ViBa}} & 0\\ 0 & m_{f,\mathrm{ViBa}} \end{array}\right]$$

composed of the mass of the ViBa, *m*_ViBa_, and the mass of its foundation, *m*_*f*,ViBa_. Also, the global stiffness matrix $\tilde{\text{K}}$ is subdivided in the following form:

2.6 $$\tilde{\text{K}}=\left[\begin{array}{ccc} \begin{array}{cc} {\tilde{\text{K}}}_{1} & {\tilde{\text{K}}}_{1,i}\\ {\tilde{\text{K}}}_{i,1} & {\tilde{\text{K}}}_{i} \end{array} & \cdots & \begin{array}{cc} {\tilde{\text{K}}}_{1,n} & {\tilde{\text{K}}}_{1,V}\\ {\tilde{\text{K}}}_{i,n} & {\tilde{\text{K}}}_{i,V} \end{array}\\ \vdots & \ddots & \vdots \\ \begin{array}{cc} {\tilde{\text{K}}}_{n,1} & {\tilde{\text{K}}}_{n,i}\\ {\tilde{\text{K}}}_{V,1} & {\tilde{\text{K}}}_{V,i} \end{array} & \cdots & \begin{array}{cc} {\tilde{\text{K}}}_{n} & {\tilde{\text{K}}}_{n,V}\\ {\tilde{\text{K}}}_{V,n} & {\tilde{\text{K}}}_{V} \end{array} \end{array}\right].$$

The main diagonal sub-matrices ${\tilde{\text{K}}}_{r}(r=1,\ldots ,n)$ of the structures to be protected are defined as (figure 2)

2.7 $${\tilde{\text{K}}}_{i}=\left[\begin{array}{cc} {\tilde{k}}_{i} & -{\tilde{k}}_{i}\\ -{\tilde{k}}_{i} & {\tilde{k}}_{i}+{\tilde{k}}_{f,i}+{\tilde{k}}_{i,V}+\sum_{\begin{array}{c} r=1\\ r\neq i \end{array}}^{n}{\tilde{k}}_{i,r} \end{array}\right].$$

Note that the complex nature of the stiffness is due to the dissipation of energy, simulated according to the hysteretic damping model given by

2.8 $$\tilde{k}=k(1+\text{i}\eta ),$$

where *η* is the loss factor and $\text{i}=\sqrt{-1}$ is the imaginary unit.

Furthermore, the matrix ${\tilde{\text{K}}}_{V}$ defines the ViBa stiffness as follows:

2.9 $${\tilde{\text{K}}}_{V}=\left[\begin{array}{cc} {\tilde{k}}_{\mathrm{ViBa}} & -{\tilde{k}}_{\mathrm{ViBa}}\\ -{\tilde{k}}_{\mathrm{ViBa}} & {\tilde{k}}_{\mathrm{ViBa}}+{\tilde{k}}_{f,\mathrm{ViBa}}+\sum_{i=1}^{n}{\tilde{k}}_{i,V} \end{array}\right].$$

Finally, the off-diagonal sub-matrices ${\tilde{\text{K}}}_{i,j}(i,j=1,\ldots ,n)$ related to the dynamic coupling between the *i*th and the *j*th structures are defined as

2.10 $${\tilde{\text{K}}}_{i,j}=\left[\begin{array}{cc} 0 & 0\\ 0 & -{\tilde{k}}_{i,j} \end{array}\right]$$

and

2.11 $${\tilde{\text{K}}}_{i,V}=\left[\begin{array}{cc} 0 & 0\\ 0 & -{\tilde{k}}_{i,V} \end{array}\right],\;{\tilde{\text{K}}}_{V,i}=\left[\begin{array}{cc} 0 & 0\\ 0 & -{\tilde{k}}_{V,i} \end{array}\right]$$

for the dynamic coupling between the *i*th structure and the ViBa. It is noted that in general the components ${\tilde{k}}_{i,V}$ and ${\tilde{k}}_{V,i}$ can be different, resulting in an asymmetric stiffness matrix.

Finally, *U*_g_ is the ground motion displacement applied to the base of each foundation and **Q** is the influence matrix that depends on the soil–foundation stiffness values as follows:

2.12 $${\text{Q}}^{T}=\left[\begin{array}{ccccccc} 0 & {\tilde{k}}_{f,i} & \cdots & 0 & {\tilde{k}}_{f,n} & 0 & {\tilde{k}}_{f,\mathrm{ViBa}} \end{array}\right].$$

It is noted that the addition of independent ground springs makes it possible to take into account ground spatial variation.

The structural parameters of the ViBa represent the unknowns of the problem, as they have to be determined in order to reduce the dynamic response of the adjacent structures. Namely, all five ViBa structural parameters, *k*_ViBa_, *m*_ViBa_, *η*_ViBa_, *k*_*f*,ViBa_, *m*_*f*,ViBa_, can be determined through an optimization procedure aimed at reducing the structural response of the adjacent buildings. However, it is noted that the relevant foundation parameters of the ViBa, namely the mass *m*_*f*,ViBa_ and the stiffness ${\tilde{k}}_{f,\mathrm{ViBa}}$, are provided through a preliminary design based on the geotechnical bearing capacity, e.g. under static load. Therefore, the optimization procedure aims to determine the remaining parameters such as the stiffness, *k*_ViBa_, the mass, *m*_ViBa_, and the damping, *η*_ViBa_, i.e. the components of the internal oscillator unit of the ViBa. These parameters are collected in the design parameters vector ***α***={*k*_ViBa_,*m*_Viba_,*η*_ViBa_}.

The objective of the ViBa is to reduce the vibrations of the adjacent structures and the consequent stresses related to the relative displacements. Therefore, the optimization problem is established as

2.13 $$\begin{array}{rl} & \min\{U_{i}^{r,\max}(\alpha )\}\;i=1,\ldots ,n,\\ & \alpha =\{k_{\mathrm{ViBa}},m_{\mathrm{ViBa}},\eta_{\mathrm{ViBa}}\}\in R_{0}^{+}, \end{array}\}$$

where $U_{i}^{r,\max}(\alpha )$ is the maximum displacement of the *i*th structure relative to its foundation:

2.14 $$U_{i}^{r,\max}=\max(U_{i}-U_{f,i}).$$

The solution of the optimization problem (2.13) is usually obtained numerically; however, closed-form expressions can be derived in some particular cases as described in the following sections.

## 3. Vibration control of two structures through the vibrating barrier

Consider a global system composed of two buildings protected by the ViBa, as illustrated in figure 2, with *i*=1 and *j*=2. The governing equation of motion of the system is

3.1 $${\tilde{\text{K}}}_{\mathrm{dyn}}(\alpha ,\omega )\text{U}(\omega )=\text{Q}U_{g}(\omega ),$$

where ${\tilde{\text{K}}}_{\mathrm{dyn}}(\alpha ,\omega )=\tilde{\text{K}}(\alpha )-\omega^{2}\text{M}(\alpha )$ is the dynamic stiffness matrix and ***α*** is the design parameters vector. If the shape of the two foundations of the buildings is identical, then the interaction with the soil is identical as well and the following relations occur: ${\tilde{k}}_{f}={\tilde{k}}_{f,1}={\tilde{k}}_{f,2}$ and ${\tilde{k}}_{\mathrm{SSSI}}={\tilde{k}}_{1,V}={\tilde{k}}_{2,V}$. Therefore, the dynamics of the problem of equation (2.1) is rewritten in the expanded form:

3.2 $$\begin{array}{rl} & \left\{[\begin{array}{cccccc} {\tilde{k}}_{1} & -{\tilde{k}}_{1} & 0 & 0 & 0 & 0\\ -{\tilde{k}}_{1} & {\tilde{k}}_{1}+{\tilde{k}}_{f}+{\tilde{k}}_{1,2}+{\tilde{k}}_{\mathrm{SSSI}} & 0 & -{\tilde{k}}_{1,2} & 0 & -{\tilde{k}}_{\mathrm{SSSI}}\\ 0 & 0 & {\tilde{k}}_{2} & -{\tilde{k}}_{2} & 0 & 0\\ 0 & -{\tilde{k}}_{1,2} & -{\tilde{k}}_{2} & {\tilde{k}}_{2}+{\tilde{k}}_{f}+{\tilde{k}}_{1,2}+{\tilde{k}}_{\mathrm{SSSI}} & 0 & -{\tilde{k}}_{\mathrm{SSSI}}\\ 0 & 0 & 0 & 0 & {\tilde{k}}_{\mathrm{ViBa}} & -{\tilde{k}}_{\mathrm{ViBa}}\\ 0 & -{\tilde{k}}_{\mathrm{SSSI}} & 0 & -{\tilde{k}}_{\mathrm{SSSI}} & -{\tilde{k}}_{\mathrm{ViBa}} & {\tilde{k}}_{\mathrm{ViBa}}+{\tilde{k}}_{f,\mathrm{ViBa}}+2{\tilde{k}}_{\mathrm{SSSI}} \end{array}\right]\\ & \;-\omega^{2}\left[\begin{array}{cccccc} m_{1} & 0 & 0 & 0 & 0 & 0\\ 0 & m_{f,1} & 0 & 0 & 0 & 0\\ 0 & 0 & m_{2} & 0 & 0 & 0\\ 0 & 0 & 0 & m_{f,2} & 0 & 0\\ 0 & 0 & 0 & 0 & m_{\mathrm{ViBa}} & 0\\ 0 & 0 & 0 & 0 & 0 & m_{f,\mathrm{ViBa}} \end{array}\right]\}\left[\begin{array}{c} U_{1}(\omega )\\ U_{f,1}(\omega )\\ U_{2}(\omega )\\ U_{f,2}(\omega )\\ U_{\mathrm{ViBa}}\\ U_{f,\mathrm{ViBa}} \end{array}\right]=\left[\begin{array}{c} 0\\ {\tilde{k}}_{f}\\ 0\\ {\tilde{k}}_{f}\\ 0\\ {\tilde{k}}_{f,\mathrm{ViBa}} \end{array}\right]U_{g}(\omega ). \end{array}$$

The above equation is analysed by resorting to the transfer function representation that provides a basis for determining the system response characteristics. The transfer functions of the system are defined as the ratio of the output **U** and input displacement *U*_g_

3.3 $$\begin{array}{rl} \text{H}(\alpha ,\omega ) & ={\tilde{\text{K}}}_{\mathrm{dyn}}^{-1}(\alpha ,\omega )\text{Q}\\ & ={\left[\begin{array}{cccccc} H_{1}(\alpha ,\omega ) & H_{f,1}(\alpha ,\omega ) & H_{2}(\alpha ,\omega ) & H_{f,2}(\alpha ,\omega ) & H_{\mathrm{ViBa}}(\alpha ,\omega ) & H_{f,\mathrm{ViBa}}(\alpha ,\omega ) \end{array}\right]}^{T}, \end{array}$$

assuming the ground motion excitation is modelled by a harmonic signal with frequency *ω*_0_. The adopted procedure consists in minimizing the transfer functions related to the structures at the input frequency *ω*_0_. Therefore, by recalling the design parameters vector ***α***={*k*_ViBa_,*m*_Viba_,*η*_ViBa_} from equation (3.4), the optimization problem is stated as

3.4 $$\begin{array}{rl} & \min\{{\text{H}}_{1}(\alpha ,\omega_{0}),H_{2}(\alpha ,\omega_{0})\},\\ & \alpha =\{k_{\mathrm{ViBa}},m_{\mathrm{ViBa}},\eta_{\mathrm{ViBa}}\}\in R_{0}^{+}. \end{array}\}$$

Clearly, the solution to the optimization problem will be straightforward if it is possible to assign a variable. It is noted that the mass of the ViBa, *m*_ViBa_, is restrained by engineering criteria (e.g. bearing capacity of the soil, volumetric restraint, etc.). Therefore, by assigning *m*_ViBa_ as a known quantity, from equation (3.4), the stiffness *k*^optimal^_ViBa_ and the damping *η*^optimal^_ViBa_ are derived in closed form by determining the zeros of the transfer functions *H*_1_(***α***,*ω*_0_) and *H*_2_(***α***,*ω*_0_) according to Den Hartog [18]. Following simple algebra, the following formula is derived:

3.5 $${\tilde{k}}_{\mathrm{ViBa}}^{\mathrm{optimal}}(\omega_{0})=\frac{\left(\omega_{0}^{2}m_{\mathrm{ViBa}})[{\tilde{k}}_{f,\mathrm{ViBa}}+{\tilde{k}}_{\mathrm{SSSI}}(2+{\tilde{k}}_{f,\mathrm{ViBa}}/{\tilde{k}}_{f})-\omega_{0}^{2}m_{f,\mathrm{ViBa}}\right]}{{\tilde{k}}_{f,\mathrm{ViBa}}+{\tilde{k}}_{\mathrm{SSSI}}(2+{\tilde{k}}_{f,\mathrm{ViBa}}/{\tilde{k}}_{f})-\omega_{0}^{2}(m_{f,\mathrm{ViBa}}+m_{\mathrm{ViBa}})}.$$

From the above equation, the stiffness $k_{\mathrm{ViBa}}^{\mathrm{optimal}}$ and the damping *η*^optimal^_ViBa_ are derived as follows:

3.6 $$\begin{array}{r} \begin{array}{cc} & k_{\mathrm{ViBa}}^{\mathrm{optimal}}=\text{Re}\left\{{\tilde{k}}_{\mathrm{ViBa}}^{\mathrm{optimal}}(\omega_{0})\right\}\\ \text{and}\; & \eta_{\mathrm{ViBa}}^{\mathrm{optimal}}=\frac{\text{Im}\left\{{\tilde{k}}_{\mathrm{ViBa}}^{\mathrm{optimal}}(\omega_{0})\right\}}{\text{Re}\left\{{\tilde{k}}_{\mathrm{ViBa}}^{\mathrm{optimal}}(\omega_{0})\right\}}, \end{array}\} \end{array}$$

where Re{⋅} and Im{⋅} indicate, respectively, the real and imaginary component of complex value ${\tilde{k}}_{\mathrm{ViBa}}^{\mathrm{optimal}}$.

Therefore, after proper tuning, the ViBa is designed for mitigating the dynamic response of both buildings.

## 4. Vibration control of a single structure through the vibrating barrier

**Figure 4.**
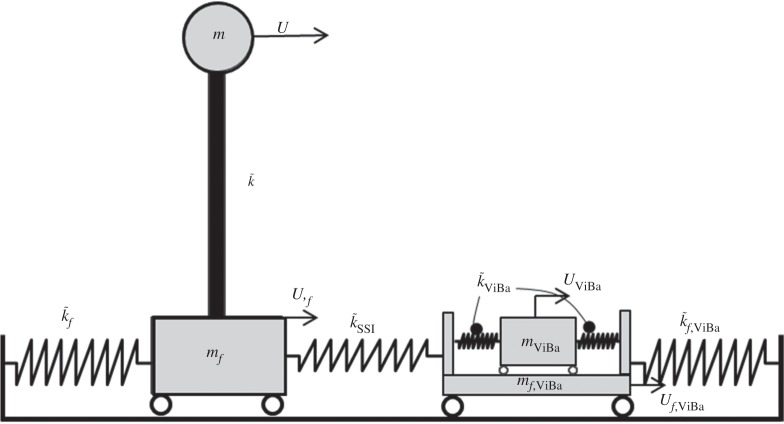
Discrete model used for the vibration control of a single structure through the ViBa.

This section describes the vibration control of a structure protected by the ViBa under harmonic base excitation. The mechanical model to be analysed is shown in figure 4. The building is modelled as a simple linear oscillator characterized by the lumped mass, *m*, and the complex stiffness, $\tilde{k};$ the oscillator is supported on a compliant restraint for simulating the SSI effects described by the complex stiffness, ${\tilde{k}}_{f}$, and the lumped mass, *m*_*f*_. The ViBa consists of the internal oscillator unit described by the lumped mass, *m*_ViBa_, and the spring complex stiffness, ${\tilde{k}}_{\mathrm{ViBa}}$, as well as of the external containment foundation idealized by the lumped mass, *m*_*f*,ViBa_, and the complex stiffness, ${\tilde{k}}_{f,\mathrm{ViBa}}$, for capturing the interaction effects with the soil. The SSSI effects are taken into account by means of a linear elastic spring, ${\tilde{k}}_{\mathrm{SSSI}}$, connecting the structure to the ViBa.

### (a) General solution

Equation (2.1) is rewritten in the expanded problem as follows:

4.1 $$\begin{array}{rl} & \left\{\left[\begin{array}{cccc} \tilde{k} & -\tilde{k} & 0 & 0\\ -\tilde{k} & \tilde{k}+{\tilde{k}}_{f}+{\tilde{k}}_{\mathrm{SSSI}} & 0 & -{\tilde{k}}_{\mathrm{SSSI}}\\ 0 & 0 & {\tilde{k}}_{\mathrm{ViBa}} & -{\tilde{k}}_{\mathrm{ViBa}}\\ 0 & -{\tilde{k}}_{\mathrm{SSSI}} & -{\tilde{k}}_{\mathrm{ViBa}} & -{\tilde{k}}_{\mathrm{ViBa}}+-{\tilde{k}}_{f,\mathrm{ViBa}}+-{\tilde{k}}_{\mathrm{SSSI}} \end{array}\right]-\omega^{2}\left[\begin{array}{cccc} m & 0 & 0 & 0\\ 0 & m_{f} & 0 & 0\\ 0 & 0 & m_{\mathrm{ViBa}} & 0\\ 0 & 0 & 0 & m_{f,\mathrm{ViBa}} \end{array}\right]\right\}\\ & \;\times \left[\begin{array}{c} U_{1}(\omega )\\ U_{f}(\omega )\\ U_{\mathrm{ViBa}}(\omega )\\ U_{f,\mathrm{ViBa}}(\omega ) \end{array}\right]=\left[\begin{array}{c} 0\\ {\tilde{k}}_{f}\\ 0\\ {\tilde{k}}_{f,\mathrm{ViBa}} \end{array}\right]U_{g}(\omega ). \end{array}$$

The transfer function is then determined as follows:

4.2 $$\begin{array}{rl} \text{H}(\alpha ,\omega ) & ={\tilde{\text{K}}}_{\mathrm{dyn}}^{-1}(\alpha ,\omega )\text{Q}\\ & ={\left[\begin{array}{cccc} H(\alpha ,\omega ) & H_{f}(\alpha ,\omega ) & H_{\mathrm{ViBa}}(\alpha ,\omega ) & H_{f,\mathrm{ViBa}}(\alpha ,\omega ) \end{array}\right]}^{T}. \end{array}$$

After simple algebra, the components of the vector **H**(***α***,*ω*) are given by

4.3 $$\begin{array}{rl} H(\alpha ,\omega ) & =\frac{U(\omega )}{U_{g}(\omega )}\\ & =\frac{\tilde{k}\cdot [({\tilde{k}}_{\mathrm{SSSI}}\cdot {\tilde{k}}_{f,\mathrm{ViBa}}+\tilde{b}\cdot {\tilde{k}}_{f})({\tilde{k}}_{\mathrm{ViBa}}-\omega^{2}m_{\mathrm{ViBa}})-{\tilde{k}}_{\mathrm{ViBa}}^{2}\cdot {\tilde{k}}_{f}]}{{\left(\tilde{k}\cdot {\tilde{k}}_{\mathrm{ViBa}}\right)}^{2}-({\tilde{k}}_{\mathrm{ViBa}}^{2}\cdot \tilde{a})(\tilde{k}-\omega^{2}m)+[(\tilde{a}\cdot \tilde{b}-{\tilde{k}}_{\mathrm{SSSI}}^{2})(\tilde{k}-\omega^{2}m)-({\tilde{k}}^{2}\cdot \tilde{b})]\tilde{c}}, \end{array}$$

4.4 $$\begin{array}{rl} H_{f}(\alpha ,\omega ) & =\frac{U_{f}(\omega )}{U_{g}(\omega )}=\left(1-\frac{\omega^{2}}{{\tilde{\omega }}^{2}}\right)H(\alpha ,\omega ), \end{array}$$

4.5 $$\begin{array}{rl} H_{\mathrm{ViBa}}(\alpha ,\omega ) & =\frac{U_{\mathrm{ViBa}}(\omega )}{U_{g}(\omega )}\\ & =\frac{{\tilde{k}}_{\mathrm{ViBa}}\cdot \{{\tilde{k}}_{f,\mathrm{ViBa}}\cdot [(\tilde{k}-\omega^{2}m)\tilde{a}-{\tilde{k}}^{2}]+{\tilde{k}}_{f}(\tilde{k}-\omega^{2}m)\cdot {\tilde{k}}_{\mathrm{SSSI}}\}}{{\left(\tilde{k}\cdot {\tilde{k}}_{\mathrm{ViBa}}^{2}\right)}^{2}-({\tilde{k}}_{\mathrm{ViBa}}^{2}\cdot \tilde{a})(\tilde{k}-\omega^{2}m)+[(\tilde{a}\cdot \tilde{b}-{\tilde{k}}_{\mathrm{SSSI}}^{2})(\tilde{k}-\omega^{2}m)-({\tilde{k}}^{2}\cdot \tilde{b})]\tilde{c}} \end{array}$$

and

4.6 $$H_{f,\mathrm{ViBa}}(\alpha ,\omega )=\frac{U_{f,\mathrm{ViBa}}(\omega )}{U_{g}(\omega )}=\left(1-\frac{\omega^{2}}{{\tilde{\omega }}_{\mathrm{ViBa}}^{2}}\right)H_{\mathrm{ViBa}}(\alpha ,\omega ),$$

where ${\tilde{\omega }}^{2}=\tilde{k}/m$ and ${\tilde{\omega }}_{\mathrm{ViBa}}^{2}={\tilde{k}}_{\mathrm{ViBa}}/m_{\mathrm{ViBa}}$; furthermore, the following positions have been made:

4.7 $$\begin{array}{rl} & \tilde{a}=\tilde{k}+{\tilde{k}}_{f}+{\tilde{k}}_{\mathrm{SSSI}}-\omega^{2}m_{f},\\ & \tilde{b}={\tilde{k}}_{\mathrm{ViBa}}+{\tilde{k}}_{f,\mathrm{ViBa}}+{\tilde{k}}_{\mathrm{SSSI}}-\omega^{2}m_{f,\mathrm{ViBa}}\\ \text{and}\; & \tilde{c}={\tilde{k}}_{\mathrm{ViBa}}-\omega^{2}{\text{m}}_{\mathrm{VIBa}}. \end{array}\}$$

The optimization problem of equation (2.13) can be restated as follows:

4.8 $$\begin{array}{rl} & \min\{H(\alpha ,\omega_{0})\},\\ & \alpha =\{k_{\mathrm{ViBa}},m_{\mathrm{ViBa}},\eta_{\mathrm{ViBa}}\}\in R_{0}^{+}. \end{array}\}$$

### (b) Closed-form solution

By assigning the mass *m*_Viba_, the optimization procedure (4.8) leads to finding the zeros of the transfer function at the frequency *ω*_0_. After some simple algebra, the following formula is derived:

4.9 $${\tilde{k}}_{\mathrm{ViBa}}^{\mathrm{optimal}}(\omega_{0})=\frac{\left(\omega_{0}^{2}m_{\mathrm{ViBa}})[{\tilde{k}}_{f,\mathrm{ViBa}}+{\tilde{k}}_{\mathrm{SSSI}}(1+{\tilde{k}}_{f,\mathrm{ViBa}}/{\tilde{k}}_{f})-\omega_{0}^{2}m_{f,\mathrm{ViBa}}\right]}{{\tilde{k}}_{f,\mathrm{ViBa}}+{\tilde{k}}_{\mathrm{SSSI}}(1+{\tilde{k}}_{f,\mathrm{ViBa}}/{\tilde{k}}_{f})-\omega_{0}^{2}(m_{f,\mathrm{ViBa}}+m_{\mathrm{ViBa}})}.$$

Also, the real and imaginary part can be separated as follows:

4.10 $${\tilde{k}}_{\mathrm{ViBa}}^{\mathrm{optimal}}(\omega_{0})=k_{\mathrm{ViBa}}^{\mathrm{real}}(\omega_{0})+\text{i}k_{\mathrm{ViBa}}^{\mathrm{imag}}(\omega_{0})=k_{\mathrm{ViBa}}^{\mathrm{optimal}}(\omega_{0})[1+\text{i}\eta_{\mathrm{ViBa}}^{\mathrm{optimal}}],$$

in which the real part $k_{\mathrm{ViBa}}^{\mathrm{real}}(\omega_{0})=k_{\mathrm{ViBa}}^{\mathrm{optimal}}(\omega_{0})$ is defined as

4.11 $$k_{\mathrm{ViBa}}^{\mathrm{real}}(\omega_{0})=\frac{\omega_{0}^{2}m_{\mathrm{ViBa}}[{\left(X^{\mathrm{real}}(\omega_{0})\right)}^{2}+{\left(X^{\mathrm{imag}}\right)}^{2}-X^{\mathrm{real}}(\omega_{0})\omega_{0}^{2}m_{\mathrm{ViBa}}]}{{\left(X^{\mathrm{real}}(\omega_{0})-\omega_{0}^{2}m_{\mathrm{ViBa}}\right)}^{2}+{\left(X^{\mathrm{imag}}\right)}^{2}},$$

where

4.12 $$\begin{array}{rl} X^{\mathrm{real}}(\omega_{0}) & =(k_{f,\mathrm{ViBa}}+k_{\mathrm{SSSI}}-\omega_{0}^{2}m_{f,\mathrm{ViBa}})\\ & \;+\;\frac{k_{\mathrm{SSSI}}k_{f,\mathrm{ViBa}}}{k_{f}(1+\eta_{f}^{2})}[1+\eta_{f}\eta_{f,\mathrm{ViBa}}+\eta_{\mathrm{SSSI}}(\eta_{f}-\eta_{f,\mathrm{ViBa}})], \end{array}$$

while the imaginary part $k_{\mathrm{ViBa}}^{\mathrm{imag}}(\omega_{0})$ is

4.13 $$k_{\mathrm{ViBa}}^{\mathrm{imag}}(\omega_{0})=\frac{-{\left(\omega_{0}^{2}m_{\mathrm{ViBa}}\right)}^{2}X^{\mathrm{imag}}}{{\left[X^{\mathrm{real}}(\omega_{0})-\omega_{0}^{2}m_{\mathrm{ViBa}}\right]}^{2}+{\left(X^{\mathrm{imag}}\right)}^{2}},$$

in which

4.14 $$\begin{array}{rl} X^{\mathrm{imag}} & =(\eta_{f,\mathrm{ViBa}}k_{f,\mathrm{ViBa}}+k_{\mathrm{SSSI}}\eta_{\mathrm{SSSI}})\\ & \;+\;\frac{k_{\mathrm{SSSI}}k_{f,\mathrm{ViBa}}}{k_{f}(1+\eta_{f}^{2})}[\eta_{\mathrm{SSSI}}(1+\eta_{\eta }\eta_{f,\mathrm{ViBa}})+\eta_{f,\mathrm{ViBa}}-\eta_{f}]. \end{array}$$

Therefore, the optimal ViBa parameters that set the structural response *U*(*ω*_0_) equal to zero, for the assigned mass *m*_ViBa_, are the following:

4.15 $$\begin{array}{rl} & k_{\mathrm{ViBa}}^{\mathrm{optimal}}=k_{\mathrm{ViBa}}^{\mathrm{real}}(\omega )\\ \text{and}\; & \eta_{\mathrm{ViBa}}^{\mathrm{optimal}}=\frac{k_{\mathrm{ViBa}}^{\mathrm{imag}}(\omega )}{k_{\mathrm{ViBa}}^{\mathrm{real}}(\omega )}, \end{array}\}$$

according to the expression given by equation (4.10).

### (c) Particular cases

It should be noted that, for lightly damping *η*≪1, the quadratic terms involving damping are negligible; thus (*X*^imag^)^2^→0 and equations (4.11) and (4.13) can be rewritten as

4.16 $$k_{\mathrm{ViBa}}^{\mathrm{real}}(\omega_{0})≅\frac{\omega_{0}^{2}m_{\mathrm{ViBa}}[{\left(X^{\mathrm{real}}(\omega_{0})\right)}^{2}-X^{\mathrm{real}}(\omega_{0})\omega_{0}^{2}m_{\mathrm{ViBa}}]}{{\left[X^{\mathrm{real}}(\omega_{0})-\omega_{0}^{2}m_{\mathrm{ViBa}}\right]}^{2}}$$

and

4.17 $$k_{\mathrm{ViBa}}^{\mathrm{imag}}(\omega_{0})≅\frac{-{\left(\omega_{0}^{2}m_{\mathrm{ViBa}}\right)}^{2}[\eta_{f,\mathrm{ViBa}}k_{f,\mathrm{ViBa}}+k_{\mathrm{SSSI}}(1+k_{f,\mathrm{ViBa}}/k_{f})(\eta_{\mathrm{SSSI}}+\eta_{f,\mathrm{ViBa}}-\eta_{f})]}{{\left[X^{\mathrm{real}}(\omega_{0})-\omega_{0}^{2}m_{\mathrm{ViBa}}\right]}^{2}}.$$

It has to be emphasized that in contrast to the common dynamic vibration absorber (DVA; e.g. [18]) in the presence of SSI effects both *k*_ViBa_ and *m*_ViBa_ have to be obtained since the ratio between the structural and soil stiffness becomes a relevant factor that must be taken into account.

In the case of undamped systems, i.e. every *η*=0, equation (4.9) reduces to

4.18 $$k_{\mathrm{ViBa}}=\frac{\left(\omega_{0}^{2}m_{\mathrm{ViBa}})[k_{f,\mathrm{ViBa}}+k_{\mathrm{SSSI}}(1+k_{f,\mathrm{ViBa}}/k_{f})-\omega_{0}^{2}m_{f,\mathrm{ViBa}}\right]}{k_{f,\mathrm{ViBa}}+{\text{V}}_{\mathrm{SSSI}}\left(1+k_{f,\mathrm{ViBa}}/k_{f}\right)-\omega_{0}^{2}(m_{f,\mathrm{ViBa}}+m_{\mathrm{ViBa}})}.$$

The calibration of the ViBa parameters according to equation (4.18) provides the total reduction of the relative displacement of the structure resulting in *U*(***α***,*ω*_0_)=0 for the harmonic excitation at a given frequency *ω*_0_.

Both the achieved formulae of equations (4.9) and (4.18) are independent from the characteristics of the above-ground structures to be protected, such as $\tilde{k}$ and *m*. Only *k*_*f*_ and *k*_SSSI_ are required for the design of the ViBa. In the case of a rigid foundation, they can be determined as a function of the foundation shape and of the stiffness of the soil. Moreover, for *m*_ViBa_≪*m*_*f*,ViBa_, equation (4.18) can be cast as

4.19 $$\omega_{\mathrm{ViBa}}^{2}=\frac{k_{\mathrm{ViBa}}}{m_{\mathrm{ViBa}}}≅\omega^{2},$$

i.e. the ViBa is tuned to the same frequency as the input signal that must be absorbed. An analogue result is determined when the coupling stiffness $k_{\mathrm{SSSI}}\to \infty$, e.g. the structure and the ViBa resting on the same rigid foundation; in this case, the governing equations derived from equations (4.3) and (4.5) are described as follows:

4.20 $$H^{\mathrm{SSSI}\to \infty }(\omega )=\frac{-k\cdot [(k_{f,\mathrm{ViBa}}+k_{f})(k_{\mathrm{ViBa}}-\omega^{2}m_{\mathrm{ViBa}})]}{\left[a(k-\omega^{2}m)-k^{2}]c+[b(k_{\mathrm{ViBa}}-\omega^{2}m_{\mathrm{ViBa}})-k_{\mathrm{ViBa}}^{2}](k-\omega^{2}m\right)}$$

and

4.21 $$H_{\mathrm{ViBa}}^{\mathrm{SSSI}\to \infty }(\omega )=\frac{-k_{\mathrm{ViBa}}\cdot [(k_{f,\mathrm{ViBa}}+k_{f})(k-\omega^{2}m)]}{\left[a(k-\omega^{2}m)-k^{2}]c+[b(k_{\mathrm{ViBa}}-\omega^{2}m_{\mathrm{ViBa}})-k_{\mathrm{ViBa}}^{2}](k-\omega^{2}m\right)},$$

where the following positions have been made: *a*=(*k*+*k*_*f*_−*ω*^2^*m*_*f*_); *b*=(*k*_ViBa_+*k*_*f*,ViBaf_−*ω*^2^*m*_*f*,ViBa_) and $c=({\tilde{k}}_{\mathrm{ViBa}}-\omega^{2}m_{\mathrm{ViBa}})$.

In this case, the ViBa is calibrated by the formula defined in equation (4.9) behaving as the classical DVA.

## 5. Numerical and experimental analyses

**Figure 5.**
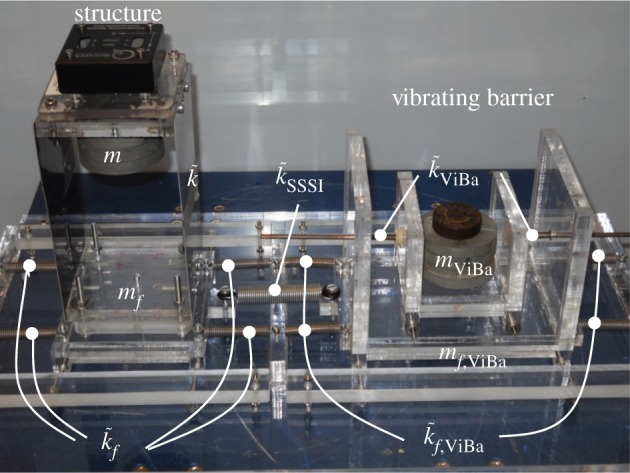
Experimental shake table set-up: prototype structure made in aluminium and acrylic connected to the shake table through elastic springs and controlled by the ViBa made of a rigid acrylic box with a 1 d.f. internal mass unit. (Online version in colour.)

In this section, numerical and experimental analyses are carried out to investigate the performance of the proposed ViBa to reduce the vibrations of structures subjected to harmonic ground motion excitation. The physical model comprises one structure to be protected coupled with a ViBa, and it is represented by the prototype in figure 5. Mechanical properties are derived directly from the prototype. Structural characteristics are reported in table 1 and in the experimental section. The first fundamental frequency of the structure by considering the compliant base is *ω*_0_=22.62 rad s^−1^. In table 2, the known mechanical characteristics of the ViBa are determined for the manufacturing of the prototype, whereas the internal device parameters described by the mass, *m*_Viba_, the stiffness, *k*_ViBa_, and the loss factor, *η*_ViBa_, are derived by the optimization procedure determined in this paper.

**Table 1.** Mechanical characteristics of the structure.

structure		
symbol	S.I.	value
*k*_1_	N m^−1^	9.0985×10^2^
*m*_1_	kg	0.590
*k*_*f*,1_	N m^−1^	640
*m*_*f*,1_	kg	0.353

**Table 2.** Mechanical characteristics of the ViBa.

ViBa		
symbol	S.I.	value
*k*_ViBa_	N m^−1^	*k*_ViBa_
*m*_ViBa_	kg	*m*_ViBa_
*k*_*f*,ViBa_	N m^−1^	760
*m*_*f*,ViBa_	kg	0.491
*k*_SSSI_	N m^−1^	315

### (a) Numerical results for harmonic base excitation

**Figure 6.**
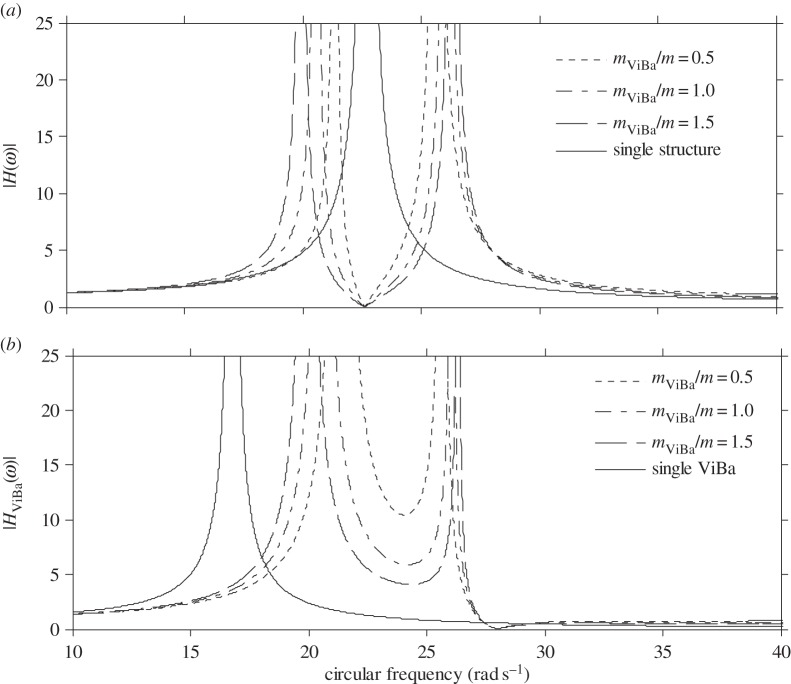
Transfer functions of the undamped system for the (*a*) structure and (*b*) ViBa obtained for different mass ratios.

**Figure 7.**
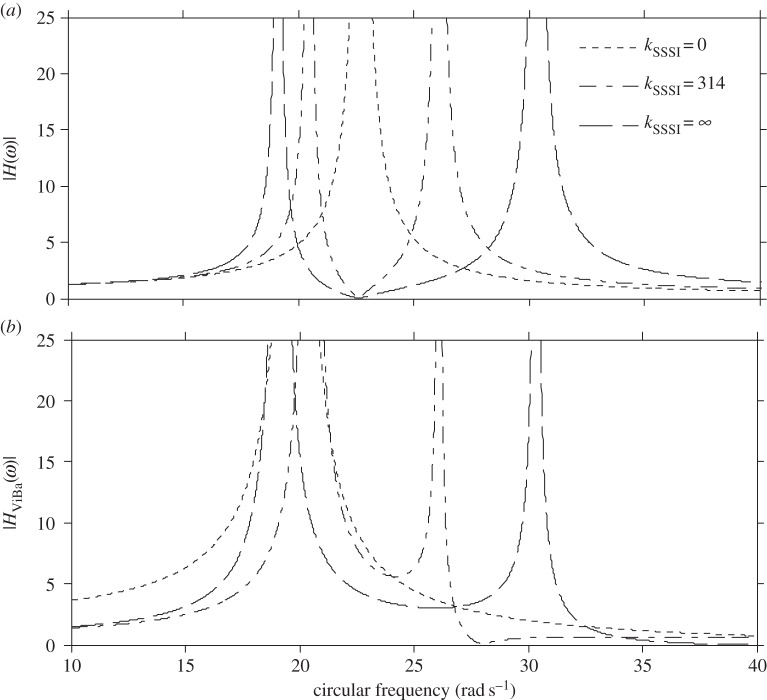
Transfer functions of the undamped system for the (*a*) structure and (*b*) ViBa obtained for different coupling stiffness values.

In this section, numerical analyses are performed to scrutinize the efficiency of the ViBa. The goal is to reduce the vibrations of the structure subjected to harmonic excitation with circular frequency *ω*_0_ equal to its first fundamental frequency, i.e. *ω*_str_=*ω*_0_=22.62 rad s^−1^, that would otherwise cause severe damage due to the induced condition of resonance. The undamped case is addressed first. Figure 6 shows the modulus of the transfer function response of the structure, |*H*(*ω*)|, and of the ViBa, |*H*_ViBa_(*ω*)|, obtained by calibrating the mass of the ViBa, *k*_ViBa_, by means of equation (4.18) for three mass ratios, *m*_ViBa_/*m*={0.5;1;1.5}, with comparison with the response of the uncoupled single structure. Each curve converges at the same null value, |*H*(*ω*)|=0, at the target circular frequency, *ω*_0_=22.62 rad s^−1^. Therefore, in undamped systems the ViBa is able to absorb 100% of the vibrations of the structure. Figure 7 depicts the modulus of the transfer function response of the structure and the ViBa obtained for different values of the coupling interaction stiffness, *k*_SSSI_. Three cases are reported, specifically the single structure when *k*_SSSI_=0, the coupled structure with ViBa when *k*_SSSI_=315 N m^−1^, i.e. the spring value used for the prototype, and the limit case of $k_{\mathrm{SSSI}}\to \infty$ (e.g. the same foundation for ViBa and the structure). The case obtained for *k*_SSSI_=0 (e.g. very long spacing between the ViBa and the structure) is identical to the uncoupled structure.

A frequency-independent hysteretic damping model for simulating the dissipation of the energy is then considered. The loss factors applied to determine the complex stiffness springs are given in table 3 and have been determined through an identification procedure.

**Table 3.** Loss factors of the global system.

symbol	value
*η*_1_	0.09
*η*_ViBa_	*η*_ViBa_
*η*_*f*,1_	0.046
*η*_*f*,ViBa_	0.04
*η*_SSSI_	0.02

Optimization of the internal parameters of the ViBa is performed by the formula described in equations (4.9)–(4.14), where the stiffness *k*^optimal^_ViBa_ is obtained once the mass *m*_ViBa_ is assigned and the optimal damping *η*^optimal^_ViBa_ is obtained by equation (4.15). It is noted that equation (4.15) yields a negative value of the loss factor *η*^optimal^_ViBa_ that cannot be adjusted since equation (4.13) is a function of only the soil and structural parameters, that is, it is independent of the parameters of the ViBa; thus the minimum real value *η*_ViBa_=0 is assigned.

**Figure 8.**
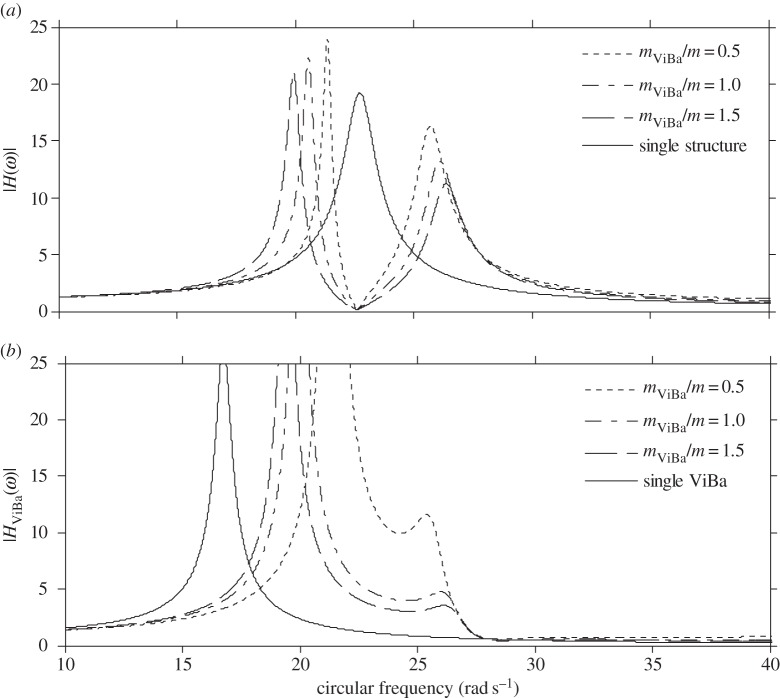
Transfer functions of the (*a*) structure and (*b*) ViBa obtained for different mass ratios and null ViBa loss factors.

 Figure 8*a* shows the modulus of the transfer function of the structural displacement, |*H*(*ω*)|, obtained by calibrating the stiffness of the ViBa, *k*_ViBa_, by means of equation (4.9) for three different mass ratios, *m*_ViBa_/*m*={0.5;1;1.5}, and compared with the response of the structure itself (referred to as a single structure). Although the steady-state responses for the three mass ratios are different because of the SSI effect, every curve converges at the same minimum value at the target circular frequency, *ω*_0_=22.62 rad s^−1^.

The achieved percentage reduction caused by the ViBa expressed in terms of the reduction factor (RF), which is defined as the ratio of the response between the coupled and uncoupled systems at the target circular frequency *ω*_0_=22.62 rad s^−1^,

5.1 $$\text{RF}=\frac{|H(\omega_{0})|}{|H^{\mathrm{uncoupled}}(\omega_{0})|},$$

is 99.05% on the structural relative displacement. On the other hand, figure 8*b* shows, for the same case, the modulus of the transfer function of the ViBa, |*H*_ViBa_(*ω*)|, showing an increment in the response with respect to the ViBa alone, manifesting the transfer of energy from the structure to the ViBa.

**Figure 9.**
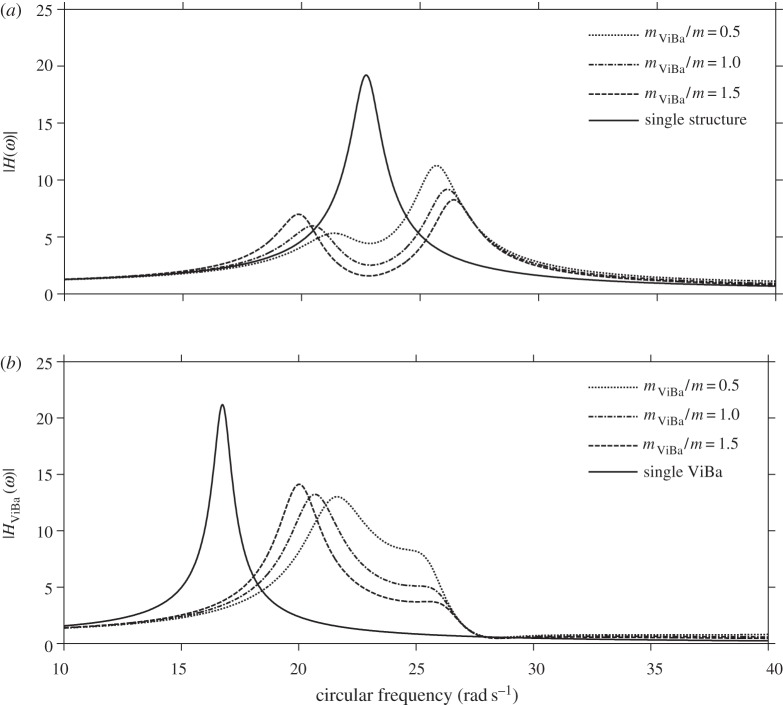
Transfer functions of the (*a*) structure and (*b*) ViBa obtained for different mass ratios and ViBa loss factor *η*_ViBa_=0.18.

It is noted that the realization of a ViBa with zero damping might be extremely challenging if not impossible. Therefore, the effect of the ViBa in the case of damping *η*^optimal^_ViBa_ different from zero is also investigated. Specifically, in addition to the previous analysis, the steady-state response is investigated by considering the loss factor experimentally measured in the prototype, i.e. *η*_ViBa_=0.18. Results are reported in figure 9. The minimum values of the response obtained at the target circular frequency, *ω*_0_=22.62 rad s^−1^, are different for the three mass ratios and are higher than the value obtained by considering the loss factor *η*_ViBa_ as null. Nevertheless, the presence of damping is fundamental to protecting the structure for a wide-band excitation. Moreover, by increasing the mass ratio, the minimum value at the target circular frequency decreases.

**Figure 10.**
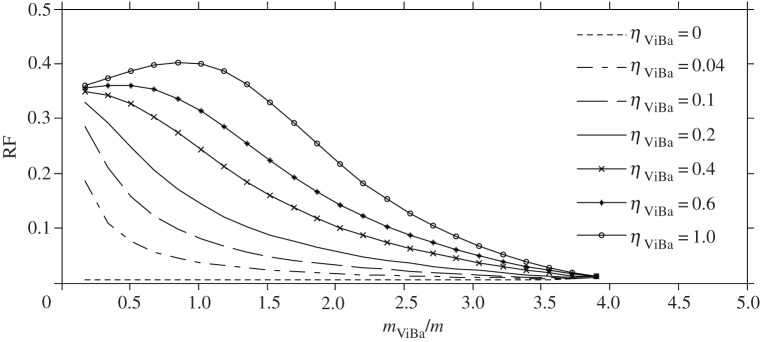
RF curves versus mass ratios for several ViBa loss factors.

The presence of damping *η*_ViBa_ different from the optimal value produces a detrimental effect for the protection of the structure subjected to harmonic loading that gets worse with an increase in the damping. This trend is illustrated in figure 10, where the RF is plotted versus the mass ratio and for various values of the loss factors *η*_ViBa_. Note that each curve lies below the unit value; therefore, the response of the structure protected by the ViBa is always smaller than the response of the same structure without protection, manifesting the efficiency of the ViBa. Also, the RF decreases with the decrease of the loss factor *η*_ViBa_ and with the increase of the mass ratio *m*_ViBa_/*m*, i.e. the lower the ViBa damping ratio, the higher the mass ratio *m*_ViBa_/*m* and the higher the reduction in the dynamic response. Moreover for very low values of the ViBa damping ratio, the RF tends to become insensitive to the mass ratio *m*_ViBa_/*m*.

### (b) Experimental test

**Figure 11.**
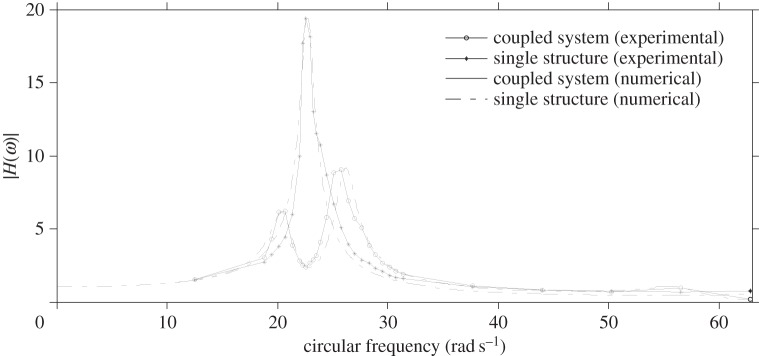
Experimentally evaluated transfer functions of the structure for the uncoupled and coupled case and comparison with numerical results.

Experimental shake table tests are performed in order to validate the analytical formulae and numerical results determined in the previous sections. The prototype as depicted in figure 5 is designed to reproduce the system of figure 4 and is tested on a 45 × 45 cm shake table. The purpose is to show the efficacy of the ViBa, calibrated by equation (4.9), in reducing the structural accelerations. The structure to be protected is realized as a one-storey shear-type building made up of acrylic for the base foundation as well as for the storey (80.5×80.5×10 mm) while aluminium sheets (80.5×141.6×0.6 mm) are used for the walls. Additional masses are placed on the top of the structures in conjunction with an accelerometer of mass 4.2 g on the top of the structure in order to record the structural response. The interaction effects with the soil are captured by linear elastic springs leading to a total stiffness *k*_*f*_=640 N m^−1^ and *k*_*f*,ViBa_=760 N m^−1^. The SSSI is represented by a linear spring *k*_SSSI_=315 N m^−1^ coupling the structure with the device. The prototype is set up on a Quanser Shake Table II for performing dynamic tests. Harmonic tests are carried out at several frequencies ranging from 2.0 to 10.0 Hz considering first the structure on its own and then coupled with the ViBa. The inherent damping is quantified through a best fit of transfer functions evaluated numerically and experimentally. Moreover, the ViBa is modelled as a rigid container with an internal oscillator made up of a spring connected to a mass and placed adjacent to the structure as shown in figure 5. The ViBa has been calibrated in order to absorb the energy affecting the structure at a selected frequency, i.e. the resonant frequency in the uncoupled system, that is, *ω*_str_=*ω*_0_=22.62 rad s^−1^. However, the device is efficient for whichever desired frequency, i.e. the device can be calibrated to absorb any target frequency. The optimal design is accomplished by means of equation (4.9). Namely, the optimal stiffness of the ViBa, calculated by means of equation (4.9), is $k_{\mathrm{ViBa}}^{\mathrm{optimal}}=440\;\text{N}\;{\text{m}}^{-1}$ for mass *m*_ViBa_ equal to 0.629 kg. The transfer function is derived as the ratio between the maximum acceleration recorded at the top of the structure and that applied to the shake table. Figure 11 shows the comparison of the calculated transfer function of the structure uncoupled and coupled with the ViBa. The experimental curves show the efficiency of the ViBa in altering the structural response. Moreover, numerical transfer function curves are drawn by means of equation (4.3), and show a good match with the experimental results. Finally, the RF calculated as the ratio between the maximum acceleration recorded in the coupled system and uncoupled system provides

$$\text{RF}=\frac{\max\{{\ddot{U}}_{1}^{\mathrm{coupled}}(t)\}}{\max\{{\ddot{U}}_{1}^{\mathrm{uncoupled}}(t)\}}=0.13.$$

**Figure 12.**
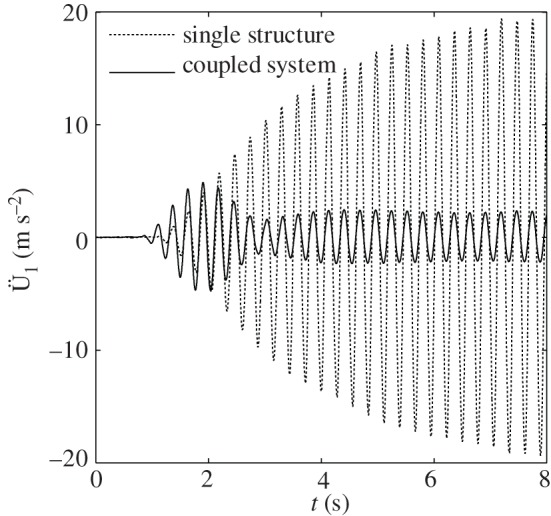
Recorded acceleration of the structure subjected to harmonic base motion at a circular frequency = 22.62 rad s^−1^ in the case of a single structure and a structure coupled with the ViBa.

Therefore, the device has been able to reduce the dynamic response of the structure by 87%. Figure 12 also reports the structural time-history accelerations for both the coupled and uncoupled case recorded during the shake table test when a harmonic base input at the target frequency *ω*_str_=*ω*_0_=22.62 rad s^−1^ with maximum acceleration of 1 m s^−2^ is applied.

### (c) Numerical application on a reactor building

**Figure 13.**
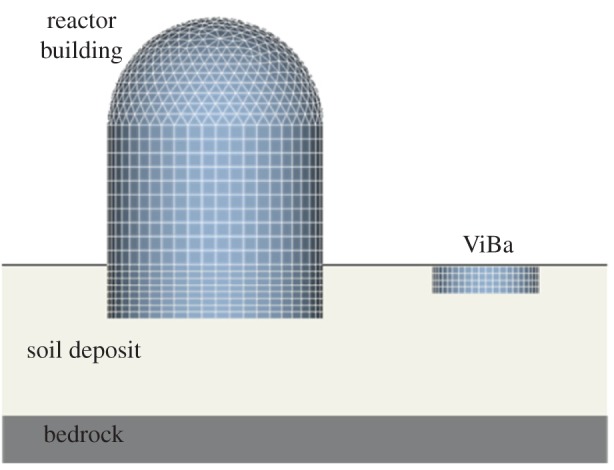
BEM/FEM model of a reactor building protected by the ViBa. (Online version in colour.)

In this section, the proposed optimization procedure for tuning the ViBa parameters is applied to investigate the response of the model of a reactor building as depicted in figure 13. The characteristics of the model of the reactor building are derived from the Electric Power Research Institute [19]. The relevant dimensions are summarized in table 4. The model of the reactor building is founded on a 30 m thick soil deposit (which is the conventional soil depth for the soil classification provided by Eurocode) characterized by shear wave velocity *V*_*s*_=400 m s^−2^, mass density *ρ*_*s*_=2.1 KN m^−3^, Poisson ratio *v*_*s*_=0.45 and hysteretic damping *η*_*s*_=0.1, resting on bedrock with a shear wave velocity of *V*_*s*_=800 m s^−2^ and hysteretic damping *η*_bed_=0.05.

**Table 4.** Basic geometry of the reactor building.

reactor building shell radius	25.8 m
basement shell radius	25.8 m
height of springline above basemat	46.12 m
embedded height	12.9 m
wall thickness	1.07 m
basemat thickness	3.05 m

The ViBa is placed at a net distance of 6.45 m in order to mitigate the response to the harmonic input with frequency *ω*_0_ equal to the first natural frequency *ω*_str_=21.05 rad s^−1^. The ViBa is modelled externally as a circular embedded foundation characterized by dimensions reported in table 5. The internal structure of the ViBa is a single oscillator characterized by the internal mass, *m*_ViBa_, the stiffness, *k*_ViBa_, and the hysteretic damping, *η*_ViBa_. The study has been undertaken by assuming linear behaviour of the structure, soil and ViBa. The substructure methodology proposed by Kausel *et al*. [20] is applied in order to solve the dynamic problem in which the reactor building is modelled according to the finite-element approach by means of the Code_Aster open source finite-element software [21], whereas the BEM formulation is used to derive the soil dynamic impedances by means of Miss3D [22]. As the BEM approach is used no internal mesh is required for the soil. The overall damping of the soil is the result of two dissipation phenomena: the first is the material damping defined by means of the complex hysteretic coefficient, the second is the radiation damping that is internally accounted for by the solution of the BEM, which applies the condition of convergence at infinity. It has to be emphasized that this approach has been validated through comparison with the experimental results of Clouteau *et al.* [23].

**Table 5.** Basic geometry of the proposed ViBa device.

basement shell radius	12.9 m
distance from reactor	12.9 m
embedded height	6.45 m
wall thickness	1.5 m
basemat thickness	1.5 m

Equation (4.9) is used for obtaining the ViBa parameters; owing to the frequency dependence of the soil dynamic impedances, the stiffness and damping values of each impedance are calculated at the circular frequency *ω*_0_. A null damping, *η*_ViBa_=0, is assigned as the optimization problem also in this case leads to an optimal negative value.

**Figure 14.**
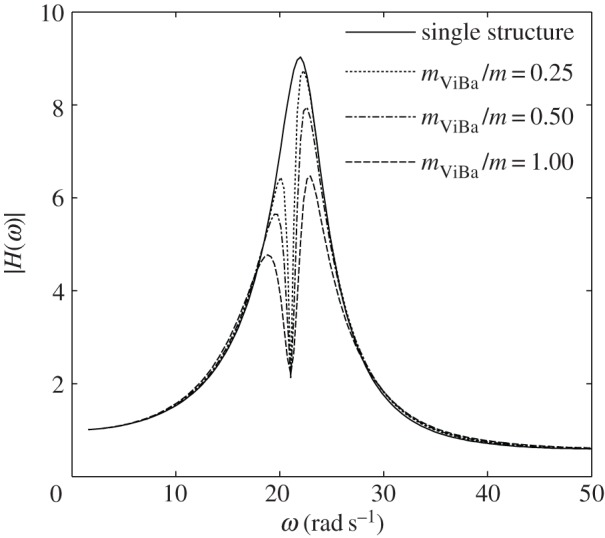
Transfer functions of the node at the top of the dome in the case of a single structure and a structure coupled with the ViBa by varying the mass ratio.

 Figure 14 shows the transfer function responses of the nodal displacement recorded at the top of the dome by varying the mass ratio between the mass, *m*_ViBa_, of the ViBa and the mass of the reactor building, *m*. Note that the reduction of the response at the circular frequency *ω*_0_ due to the effect of coupling with the ViBa is independent from the mass ratio, as already shown in figure 8 in the case of null ViBa damping. The calculated RF is 0.25; therefore, a relevant reduction of 75% of the absolute displacement is achieved. This shows the efficiency of the ViBa in realistic cases by designing the device with formulae determined through simplified structural models.

**Figure 15.**
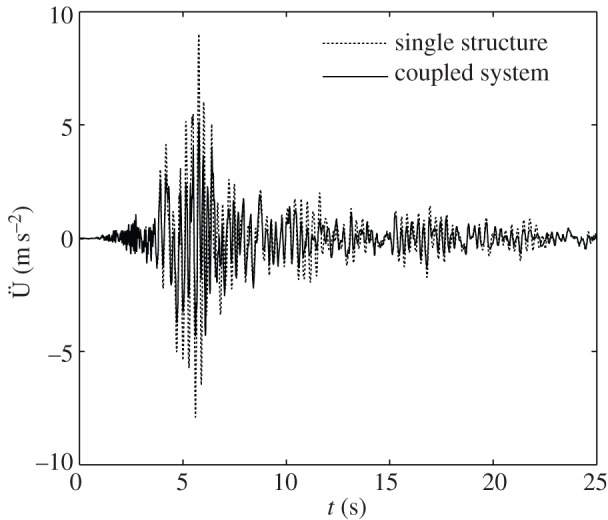
Time-history acceleration response of the node at the top of the dome in the case of a single structure and a structure coupled with the ViBa for the 1989 Loma Prieta earthquake event.

Finally, the performance of the ViBa is tested for a wide-band signal. The real ground motion recorded in the 1989 Loma Prieta earthquake event is thus applied to the system. The ViBa is designed by equation (4.9) by considering the mass ratio *m*_ViBa_/*m*=1.5. Figure 15 shows the comparison of the responses in terms of the acceleration at the top of the dome for both cases of a single structure and a structure protected by the ViBa. The comparison of the time-history accelerations show a beneficial effect due to the ViBa achieving a reduction in the maximum acceleration of 43.2% (RF=0.568).

## 6. Conclusion

A novel passive control device, called ViBa, is herein presented. The ViBa aims to reduce the vibrations of a cluster of neighbouring structures, subjected to ground motion waves, by exploiting the SSSI effect. The proposed vibration control strategy presents a novel approach moving towards a global solution for the passive control of structures under ground motion as an alternative to the conventional localized solutions.

A simplified discrete model has been determined to analyse the performance of the proposed device on the structural dynamic response. Closed-form solutions have been derived for designing the ViBa in order to protect surrounding buildings subjected to harmonic ground motion waves.

Numerical analyses are performed to evaluate the performance of the ViBa calibrated through the proposed procedure. Results have confirmed the efficiency of the proposed closed-form solutions. The ideal condition of zero damping has been considered first to study the performance of the ViBa, and for this academic case a reduction of 100% of the relative structural displacement has been shown. For the damped condition in all the cases analysed, a reduction greater than 75% has been found.

Shake table experimental tests have been performed showing a significant reduction in the structural acceleration of 87%.

It has to be emphasized that, even if the ViBa has been designed to control harmonic excitations, it was able to significantly reduce the response of a nuclear reactor to broad-band excitation as well.

Clearly, it has to be pointed out that all the three seismic wave components cannot be damped by a single one-dimensional ViBa. To this aim, a three-dimensional ViBa should be used. Also in analogy with the tuned mass damper (TMD) technology, multiple ViBas might also be embedded in the soil to take account of a large variety of inputs.

In addition, the paper addresses the simplistic hypothesis of linear soil behaviour and nonlinearity might induce issues in the tuning of the ViBa. A possible approach to consider nonlinear soil behaviour in the study is to use the formulation presented in this paper by considering nonlinear springs as proposed in Allotey & El Naggar [24] and then calibrate the ViBa numerically.

The major criticism that can be raised for the novel device is the large mass of the ViBa. Although the basic working principle is similar to TMD systems, the mass of the ViBa needs to be large in comparison with the TMD. It has to be emphasized, on the other hand, that the ViBa can be designed to reduce the vibrations of more than one building and/or for buildings of historical relevance, for which it might still be considered a viable solution.
